# Comparison of myocardial fibrosis quantification methods by cardiovascular magnetic resonance imaging for risk stratification of patients with suspected myocarditis

**DOI:** 10.1186/s12968-019-0520-0

**Published:** 2019-02-28

**Authors:** Christoph Gräni, Christian Eichhorn, Loïc Bière, Kyoichi Kaneko, Venkatesh L. Murthy, Vikram Agarwal, Ayaz Aghayev, Michael Steigner, Ron Blankstein, Michael Jerosch-Herold, Raymond Y. Kwong

**Affiliations:** 1 Noninvasive Cardiovascular Imaging, Cardiovascular Division, Department of Medicine, Brigham and Women’s Hospital, Harvard Medical School, 75 Francis Street, Boston, MA 02115 USA; 2 Cardiovascular Imaging, Department of Radiology, Frankel Cardiovascular Center, University of Michigan, Ann Arbor, MI USA; 3 Noninvasive Cardiovascular Imaging, Department of Radiology, Brigham and Women’s Hospital, Harvard Medical School, Boston, MA USA; 4 Cardiac Magnetic Resonance Imaging, Cardiovascular Division, Department of Medicine, Brigham and Women’s Hospital, Harvard Medical School, 75 Francis Street, Boston, MA 02115 USA

**Keywords:** Myocarditis, Outcome, MACE, CMR, Cardiovascular magnetic resonance imaging, Quantification method, Full width half maximum, FWHM, Standard deviation, SD

## Abstract

**Background:**

Although the presence of late gadolinium enhancement (LGE) using cardiovascular magnetic resonance imaging (CMR) is a significant discriminator of events in patients with suspected myocarditis, no data are available on the optimal LGE quantification method.

**Methods:**

Six hundred seventy consecutive patients (48 ± 16 years, 59% male) with suspected myocarditis were enrolled between 2002 and 2015. We performed LGE quantitation using seven different signal intensity thresholding methods based either on 2, 3, 4, 5, 6, 7 standard deviations (SD) above remote myocardium or full width at half maximum (FWHM). In addition, a LGE visual presence score (LGE-VPS) (LGE present/absent in each segment) was assessed. For each of these methods, the strength of association of LGE results with major adverse cardiac events (MACE) was determined. Inter-and intra-rater variability using intraclass-correlation coefficient (ICC) was performed for all methods.

**Results:**

Ninety-eight (15%) patients experienced a MACE at a medium follow-up of 4.7 years. LGE quantification by FWHM, 2- and 3-SD demonstrated univariable association with MACE (hazard ratio [HR] 1.05, 95% confidence interval [CI]:1.02–1.08, *p* = 0.001; HR 1.02, 95%CI:1.00–1.04; *p* = 0.001; HR 1.02, 95%CI: 1.00–1.05, *p* = 0.035, respectively), whereas 4-SD through 7-SD methods did not reach significant association. LGE-VPS also demonstrated association with MACE (HR 1.09, 95%CI: 1.04–1.15, *p* < 0.001). In the multivariable model, FWHM, 2-SD methods, and LGE-VPS each demonstrated significant association with MACE adjusted to age, sex, BMI and LVEF (adjusted HR of 1.04, 1.02, and 1.07; *p* = 0.009, *p* = 0.035; and *p* = 0.005, respectively). In these, FWHM and LGE-VPS had the highest degrees of inter and intra-rater reproducibility based on their high ICC values.

**Conclusions:**

FWHM is the optimal semi-automated quantification method in risk-stratifying patients with suspected myocarditis, demonstrating the strongest association with MACE and the highest technical consistency. Visual LGE scoring is a reliable alternative method and is associated with a comparable association with MACE and reproducibility in these patients.

**Trial registration number:**

NCT03470571. Registered 13th March 2018. Retrospectively registered.

## Background

Myocarditis is a frequent cause of dilated cardiomyopathy [1]. Myocarditis has diverse pattern of clinical signs and symptoms at presentations [2, 3] and its diagnosis may be challenging, as the sensitivity of the main diagnostic tools may vary greatly [1, 4, 5]. Cardiovascular magnetic resonance imaging (CMR) has become the primary imaging tool for establishing the diagnosis by the use of late gadolinium enhancement (LGE) imaging [2, 5–8]. We recently showed an incremental prognostic value of LGE in risk stratifying patients with suspected myocarditis [9]. However, an optimal method of LGE quantification in patients with suspected myocarditis is currently unknown. Better characterization of LGE pattern is important given that the heterogeneity in presence, localization and intensity of LGE extent in myocarditis [6] and that may improve clinical decisions. We therefore sought to compare LGE quantification techniques including thresholding by 2, 3, 4, 5, 6 or 7 standard deviations (SDs) above remote myocardium, the full width at half maximum (FWHM) technique, and visual quantification, as well as their respective association with clinical outcome in a post-hoc analysis of patients with suspected myocarditis [9].

## Methods

### Study population

The study included consecutive patients referred for CMR at our center for “suspected myocarditis” as the primary clinical question between December 2002 and December 2015 [9]. In real-world clinical practice, diagnosis of myocarditis is challenged by a lack of reference standards including the use of noninvasive measures, serum biomarkers, and even tissue pathology from invasive biopsies [10]. We included consecutive patients with suspected myocarditis raised as a referral indication with presenting signs/symptoms from either one of 2 groups: 1) acute chest pain syndromes with symptom onset < 2 weeks before CMR; 2) subacute (onset ≥2 weeks) of dyspnea, signs of left ventricular (LV) dysfunction, or ventricular arrhythmias syncopal spells or abnormal electrocardiogram (ECG). Exclusion criteria included: 1) any evidence of coronary artery disease (CAD) by either previous documented medical history, any previous or newly detected relevant CAD in non-invasive or invasive imaging or subendocardial LGE consistent of CAD in a territory subtended by a coronary vessel 2) any evidence of hypertrophic cardiomyopathy, arrhythmogenic right ventricular (RV) cardiomyopathy, cardiac sarcoidosis, or cardiac amyloidosis; and 3) any evidence of Takotsubo cardiomyopathy, constrictive pericarditis, Loeffler endocarditis, ventricular noncompaction, cardiac tumor, pulmonary embolism, or severe valvular disease. Seven hundred forty-four patients were included. Fifty-nine (7.9%) patients were excluded based on CMR findings consistent with: myocardial infarction (*N* = 35), biopsy-proven cardiac amyloidosis (*N* = 6), ventricular non-compaction (N = 3), Takotsubo cardiomyopathy (*N* = 4), constrictive pericarditis (*N* = 2), cardiac sarcoidosis (N = 2), Loeffler endocarditis (N = 2), and 1 each for arrhythmogenic cardiomyopathy, hypertrophic cardiomyopathy, pulmonary embolism, cardiac tumor, and severe valvular disease. Fifteen patients were excluded due to technical reasons (claustrophobia with incomplete CMR scans or non-diagnostic LGE images) [9]. Takotsubo cardiomyopathy was defined as previously published with apical ballooning [11], elevated troponin, absence of CMR features suggesting of myocarditis (absence of LGE) and absence of coronary artery disease. Clinical data including medication, laboratory tests including cardiac biomarkers, and ECG before CMR scanning were analyzed. Abnormal troponin I was defined < 0.10 ng/mL and troponin *T* < 14 ng/l. Normal values of creatine phosphokinase (CPK) were < 145 U/l in women and < 170 U/l in men. Clinical data, cardiac biomarkers and ECG were analyzed at baseline by a cardiologist.

### CMR imaging protocol and image post-processing

The CMR systems included a 3T (Tim Trio, Siemens Healthineers, Erlangen, Germany) and a 1.5 T (Aera, Siemens Healthineers). All patients underwent cine balanced steady-state free precession (bSSFP) imaging and an LGE imaging protocol (TR, 4.8 ms; TE, 1.3 ms; inversion time, 200 to 300 ms), using a segmented inversion-recovery pulse sequence starting 10 to 15 min after a weight-based injection (cumulative dose 0.15 mmol/Kg) of gadolinium diethylenetriamine pentaacetic acid (Magnevist, Bayer HealthCare Pharmaceuticals Inc., Berlin, Germany). In 122 (21%) patients, Multihance (Bracco Diagnostic, Milan, Italy) was used instead of Magnevist. In patients with an estimated glomerular filtration rate (eGFR) < 60 mL/min/1.73m^2^, contrast dose was restricted to 0.1 mmol/kg or 20 ml, whichever was lower in volume in compliance with our institutional policy [12]. A commercially available software (MASS v15, Medis, Leiden, The Netherlands) was used to post-process and quantify all CMR images.

### LGE quantification

**Fig. 1:**
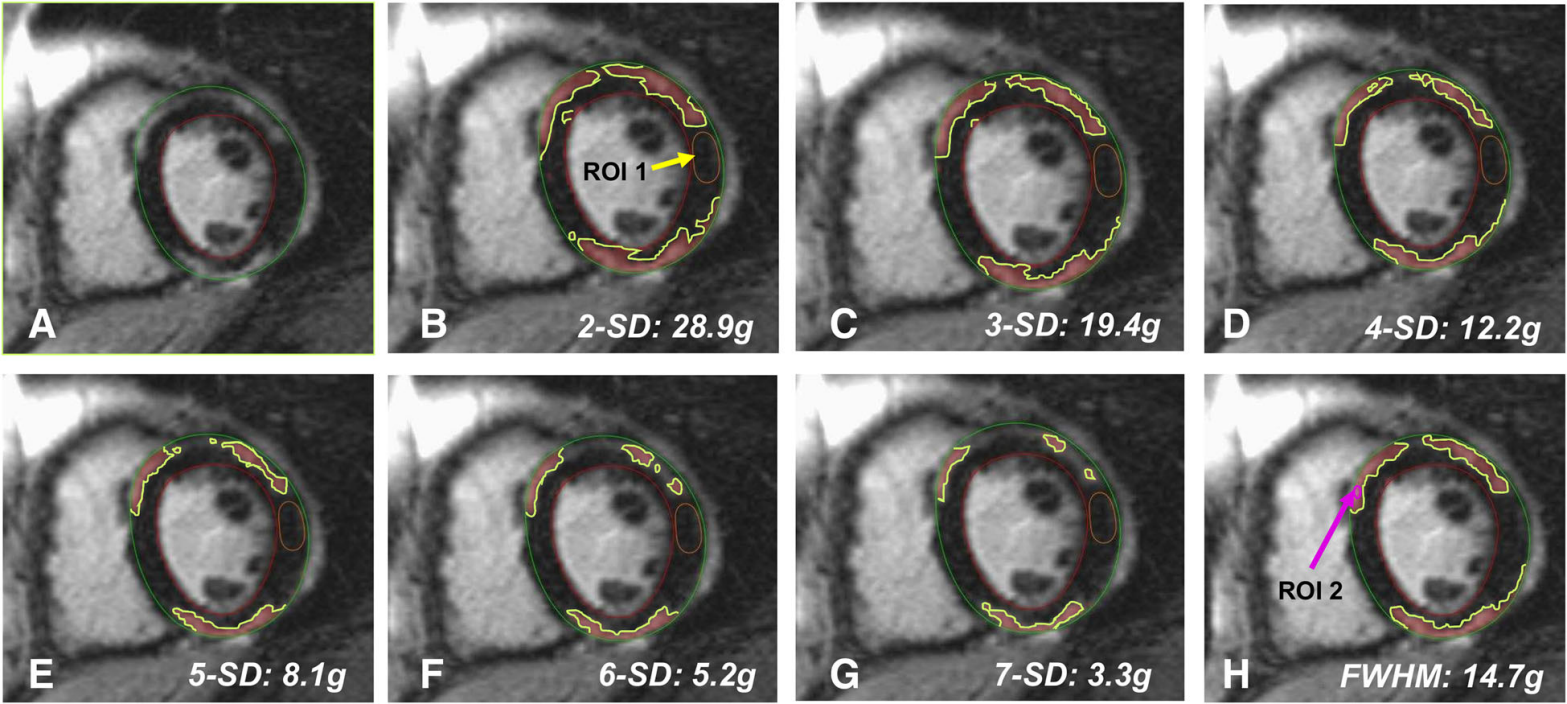
Example of the different late gadolinium enhancement (LGE) quantification methods in a patient with suspected myocarditis. **a** LGE-image with endocardium and epicardium is demarcated, **b** 2-SD (LGE: 28.9 g, 24.9% of total left ventricular (LV) mass); **c** 3-SD (19.4g, 16.8%); **d** 4-SD (12.2 g, 10.5%); **e** 5-SD (8.1g, 7.0%); **f** 6-SD (5.2 g, 4.5%); **g** 7-SD (3.3 g, 2.9%); **h** full width half maximum (FWHM) (14.7 g, 12.6%). Total LV mass was 116 g. The fibrosis is outlined in yellow. For 2 to 7-SD a region of interest (ROI) 1 is identified in the reference remote myocardium (yellow arrow/yellow contour). For FWHM, an automated ROI 2 is identified in the affected myocardium (pink arrow/pink contour). Of note, only the midventricular slice is represented, however, total LGE quantification includes mass and percentage of the entire left ventricle. SD = standard deviation

Semi-automated quantification was performed as follows: Epicardial and endocardial LV contours were carefully placed manually on all LGE images. The remote non-LGE reference region of each LGE slice was placed adjacent to the region of LGE so that the reference region is at approximate equal distance from the anterior receiver coils. Therefore, we believe this method minimizes any modifying effect from LGE location to robustness of the LGE quantitation. LGE mass was then quantified by semi-automatic methods using a signal intensity threshold of > 2,3,4,5,6,7-SD, respectively above a reference region of remote myocardium (adjacent to the region of LGE and approximately equal in distance to anterior receiver coils) in the same slice, and using regions defined as above 50% of maximal signal intensity of the enhanced area for the FWHM approach (see Fig. 1) [13, 14]. Artifacts were manually erased. In all methods LGE mass (in grams), was then expressed as a percentage of total LV mass determined by bSSFP cine images [15]. LGE extent was also determined visually by the 17 segments model by using two different scores: [1] LGE being present or not in the segment defined the visual presence score (LGE-VPS) (maximum score 17), and [2] the visual transmurality score (LGE-VTS) summed the transmural extent of LGE per segment, assessed by a five-point scale (0 = no LGE, 1 = < 25% transmurality, 2 = 26–50% transmurality, 3 = 51–75% transmurality, 4 = 76–100% transmurality) (maximum score 68). VPS sizing aimed to include any abnormal enhancement on LGE images including regions of intermediate signal intensity. LGE image quality was graded as: 1 = poor image quality, 2 = fair image quality, 3 = good image quality, and 4 = excellent image quality. LGE images were evaluated by the consensus of two American College of Cardiology Core Cardiovascular Training Statement (COCATS) level III experienced readers (CG and RYK) and inter-rater reproducibility testing was performed by an independent experienced CMR investigator (KK).

### Follow-up of clinical endpoints

We reviewed all available electronic medical records including mortality status from Social Security Death Index for all subjects. Subjects were then sent a standardized checklist-based patient-questionnaire by mail and/or followed-up by a scripted telephone interview based on the same standardized checklist 2 weeks later. Subjects were given an option to refuse to be contacted by telephone whether or not they decided to return the questionnaire by mail. Subjects who we were not able to establish contact for at least 6 months after the CMR were considered lost to follow-up. We defined the primary endpoint being a composite major adverse cardiac events (MACE): a) all-cause death, heart failure decompensation requiring hospital admission as defined in prior trials [16, 17], heart transplantation, documented sustained ventricular arrhythmia (> 30 s), recurrent acute myocarditis based on clinical biomarkers and CMR Lake-Louise definition [6]. Time-to-MACE was determined from the CMR study date to the occurrence of the first MACE or censorship at end of the follow-up period. All study procedures were reviewed and approved by our Institutional Review Board in accordance with institutional guidelines.

### Statistical analysis

Categorical variables were presented as percentages of the entire cohort or as percentage of the corresponding group if relevant data were missing. Continuous variables were expressed as mean ± standard deviation or as median values with interquartile range [IQR] depending on normality of distributions. Categorical variables were compared using the Chi^2^ or Fisher exact test in low field numbers, whereas comparisons for continuous data were performed using 2-sample Student t-test or Wilcoxon rank-sum test, when appropriate. A two-sided *p*-value of < 0.05 was deemed significant. Univariable associations with MACE and chi-square were determined by Cox proportional hazards regression. Multivariable (simultaneous entry, i.e. enter method) associations of risk covariates with MACE were determined by Cox proportional hazards regression. Multivariable models were built by including LGE extent from the various quantification methods and clinical variables in order to test for the prognostic association of LGE extent incremental to common clinical risk markers. For each multivariable model, the assumption of proportional hazards was checked and confirmed valid. For inter-rater and intra-rater reliability analyses, we randomly selected 20 patients with LGE presence and compared the measurement of LGE extent using all 7 quantitative methods (2-SD through 7-SD, FWHM) by two independent readers. Each CMR scan was independently contoured by the 2 readers and automated region of reference (ROI) was selected to generate the LGE extents for comparison. Furthermore, the LGE-VPS and LGE-VTS visual scores by two independent readers were assessed for Intra- and inter-reader agreement using intraclass-correlation coefficient (ICC). SAS (version 9.4, SAS Institute Inc., Cary, USA) and SPSS 22.0 (International Business Machines Corporation, Armonk, New York, USA) software packages were used for analysis.

## Results

**Fig. 2:**
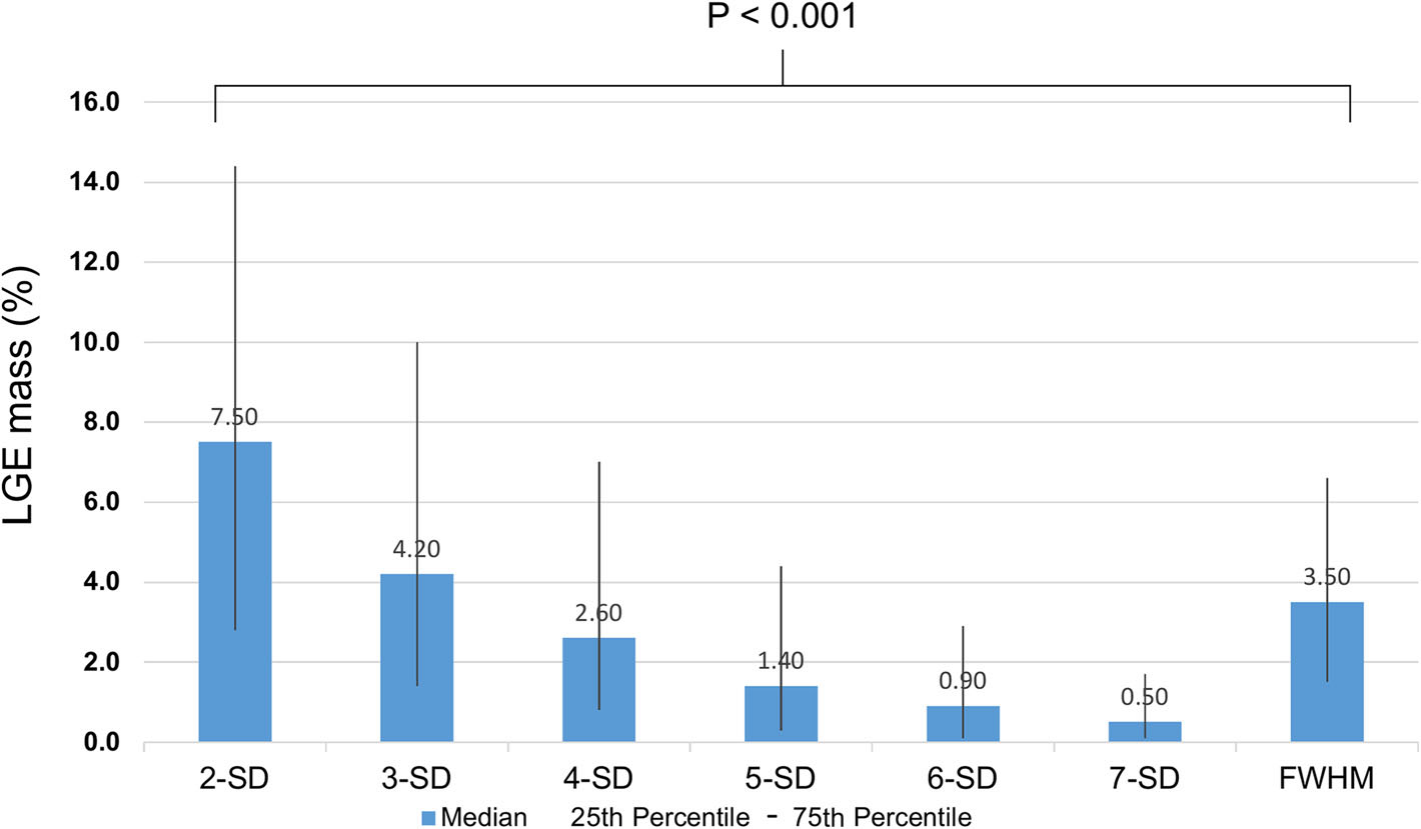
Difference in LGE mass (%) between different semi-automated quantification methods in LGE positive cases are displayed. Comparing the different semi-quantitative LGE quantification methods, the greatest amount of LGE was measured with the 2-SD method and lowest with the 7-SD method. Confidence intervals were broader in lower SD methods. LGE = Late gadolinium enhancement, FWHM = Full width at half maximum, SD = Standard deviation

A total of 670 patients represented our study group with 2 (0.3%) patients lost to follow-up and a median follow-up of 4.7 [IQR 2.3–7.3] years. Mean age was 48 ± 16 years and 392 (59%) patients were male. CMR studies were performed using a 3 T scanner in 535 (80%) patients and the remaining using a 1.5 T scanner. 350 (52%) patients presented with acute chest pain syndromes (< 2 weeks) and the remaining 320 (48%) were subacute presentations (> 2 weeks). LGE was present in 294 (44%) patients. Among these 169 (57%) were in the acute presentation group and 125 (43%) were in the subacute presentation group. Baseline and CMR characteristics are depicted in Tables 1 and 2. In 24 (3.5%) patients, LGE image quality was poor, in 92 (13.7%) image quality was fair, in 356 (53.1% image quality was good and in 198 (29.6%) image quality was excellent. LGE extent is presented in Fig. 2.

**Table 1:** Baseline Characteristics

	All patients (*n* = 670)
*Baseline*	
Age (year)	47.8 ± 16.0
Female sex	278 (41%)
BMI, kg/m^2^	27.8 ± 6.3
*Acuteness of Presentation*	
Acute chest pain syndromes (< 2 weeks)	350 (52%)
Subacute presentation (> 2 weeks) with dyspnea or left ventricular dysfunction	201 (30%)
Subacute presentation (> 2 weeks) with ventricular arrhythmias, syncopal spells or abnormal ECG	119 (18%)
*Prior Cardiovascular History*	
Hypertension	181 (27%)
Tobacco	76 (11%)
Diabetes	60 (9%)
Dyslipidemia	138 (21%)
*Medications*	
Aspirin	186 (28%)
ACE inhibitors	229 (35%)
Beta-blockers	266 (40%)
Diuretics	135 (21%)
Statins	142 (22%)
Insulin	23 (4%)
*ECG*	
Abnormal ECG	278 (42%)
*Laboratory Testing’s*	
Troponin abnormal	170 (63%)
Creatine-kinase abnormal	70 (40%)
White blood cell count abnormal	105 (35%)

*ACE* angiotensin converting enzyme inhibitor, *BMI* body mass index, *ECG* electrocardiogram

**Table 2:** CMR Baseline Characteristics

	All patients (*n* = 670)
LVEF (%)	50 ± 15
LVEDVi (ml/m^2^)	98 ± 33
LVESVi (ml/m^2^)	53 ± 34
LV mass index (g/m^2^)	61 ± 17
RVEF (%)	49 ± 11
RVEDVi (ml/m^2^)	80 ± 21
RVESVi (ml/m^2^)	42 ± 17
Pericardial effusion	169 (25%)
T2- weighted imaging (SIR ≥2)	124 (27%)
LGE presence	292 (44%)
LGE-VPS	1.7 ± 3.4
LGE-VTS	4.2 ± 8.9
LGE mass (g)	
- 2-SD	5.5 ± 10.6
- 3-SD	3.7 ± 7.7
- 4-SD	2.6 ± 5.8
- 5-SD	1.7 ± 4.1
- 6-SD	1.2 ± 3.2
- 7-SD	0.8 ± 2.4
- FWHM	2.7 ± 5.3
LGE mass (%)	
- 2-SD	4.7 ± 8.8
- 3-SD	3.1 ± 6.5
- 4-SD	2.2 ± 4.9
- 5-SD	1.5 ± 3.5
- 6-SD	1.0 ± 2.7
- 7-SD	0.7 ± 2.1
- FWHM	2.2 ± 4.5

*CMR* cardiovascular magnetic resonance imaging, *LVEF* left ventricular ejection fraction, *LVEDVi* left ventricular end diastolic volume indexed, *LVESVi* left ventricular end systolic volume index, *RVEF* right ventricular ejection fraction, *RVEDVi* right ventricular end-diastolic volume index, *RVESVi* right ventricular end-systolic volume index, *LGE* late gadolinium enhancement,. *SIR* signal intensity ratio (ratio of signal in myocardium divided by signal in skeletal muscle), *VPS* visual presence score, *VTS* visual transmurality score, *FWHM* full width half maximum;

T2 weighted imaging is available in 465 patients

### LGE extent and outcome

**Fig. 3:**
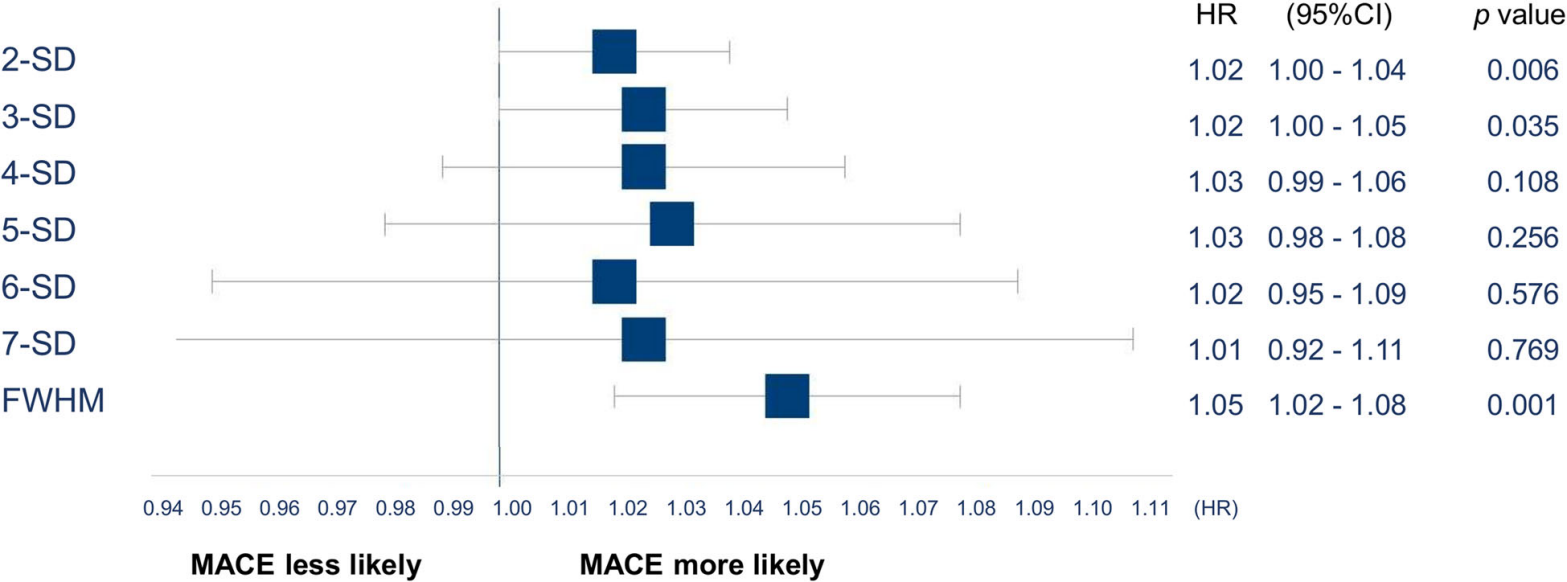
Univariable association of different semi-automated LGE (%) quantification methods with outcome. Comparing the different semi-quantitative LGE quantification methods, only 2-SD, 3-SD and FWHM were significantly associated with MACE. LGE = Late gadolinium enhancement, FWHM = Full width at half maximum, SD = Standard deviation, MACE = Major adverse cardiovascular event, HR = Hazard ratio, CI = Confidence interval

Ninety-eight (15%) patients showed a MACE at follow-up. MACE included 29 deaths (4%), 38 heart failure hospitalizations (6%), 22 sustained ventricular arrhythmias (3%), 7 recurrent myocarditis (1%), and 2 cases of heart transplantation (0.3%). As depicted in Fig. 3, LGE (%) quantification by 2-, 3-SD or FWHM demonstrated robust prognostic association with MACE, whereas 4-SD through 7-SD showed non-significant prognostic association. Association of clinical parameter, CMR characteristics and different LGE extent (g) with outcome are depicted in Table 3.

**Table 3:** Univariable Association of Clinical and CMR for MACE

	MACE	
Potential Predictors	HR (95% CI)	*p* value
*Baseline*		
Age (year)	1.03 (1.01–1.04)	< 0.001
Female sex	1.60 (1.07–2.38)	0.021
Body mass index (kg/m^2^)	1.05 (1.02–1.08)	0.001
*Referral reasons*		
Acute (< 2 weeks) vs. subacute presentation (≥2 weeks)	1.87 (1.22–2.86)	0.003
*History*		
Hypertension	1.72 (1.14–2.61)	0.011
Tobacco	1.59 (0.99–2.58)	0.057
Diabetes	2.51 (1.49–4.22)	0.001
Dyslipidemia	1.46 (0.93–2.28)	0.101
*Medications*		
Aspirin	1.47 (1.19–1.82)	< 0.001
ACE inhibitors	1.80 (1.21–2.68)	0.004
Beta-blockers	2.34 (1.55–3.51)	< 0.001
Diuretics	3.03 (2.01–4.56)	< 0.001
Statins	1.50 (0.95–2.35)	0.080
Insulin	3.62 (1.82–7.21)	< 0.001
*ECG*		
Abnormal ECG	1.16 (0.78–1.74)	0.455
*Laboratory Testing’s*		
Troponin abnormal	1.01 (0.57–1.79)	0.968
Creatine-kinase abnormal	1.19 (0.62–2.29)	0.596
White blood cell count abnormal	1.79 (1.09–2.92)	0.021
*CMR characteristics*		
LVEF (%)	0.95 (0.94–0.97)	< 0.001
LVEDVi (ml/m^2^)	1.01 (1.00–1.02)	< 0.001
LVESVi (ml/m^2^)	1.01 (1.01–1.02)	< 0.001
LV mass index (g/m^2^)	1.01 (1.00–1.03)	0.021
RVEF (%)	0.95 (0.93–0.96)	< 0.001
RVEDVi (ml/m^2^)	1.00 (0.99–1.01)	0.570
RVESVi (ml/m^2^)	1.02 (1.01–1.03)	< 0.001
Pericardial effusion	2.31 (1.54–3.45)	< 0.001
T2- weighted imaging (SIR ≥2)	2.14 (1.30–3.52)	0.003
*CMR LGE quantifications methods*		
LGE mass (g)		
- 2-SD	1.02 (1.00–1.03)	0.011
- 3-SD	1.02 (0.99–1.04)	0.064
- 4-SD	1.02 (0.99–1.05)	0.162
- 5-SD	1.01 (0.97–1.06)	0.356
- 6-SD	1.01 (0.95–1.07)	0.754
- 7-SD	1.00 (0.92–1.09)	0.973
- FWHM	1.04 (1.02–1.06)	0.001
*CMR LGE visual assessment*		
LGE presence	2.22 (1.47–3.35)	< 0.001
LGE-VPS	1.09 (1.04–1.15)	< 0.001
LGE-VTS	1.02 (1.01–1.04)	< 0.001

*CMR* cardiac magnetic resonance imaging, *LGE* Late gadolinium enhancement, *HR* Hazard ratio, *SD* Standard deviation, *FWHM* Full width half maximum, *MACE* Major adverse cardiac events, *VPS* visual presence score, *VTS* visual transmurality score

By univariable analysis of LGE-VPS, a 9% increase in MACE hazards was observed per each abnormal LGE segment (HR 1.09, 95%CI: 1.04–1.15, *p* < 0.001). LGE-VTS showed a HR of 1.02 (95%CI: 1.01–1.04, *p* < 0.001). Age, female sex and body mass index (BMI) were associated with MACE (HR 1.03, 95%CI: 1.01–1.04, *p* < 0.001; HR 1.60, 95%CI: 1.07–2.38, *p* = 0.021 and HR 1.05, 95%CI 1.02–1.08, *p* = 0.001, respectively). When re-analyses were performed for death, heart failure hospitalization, and a need for heart transplant, LGE presence maintained robust association with these composite events, whereas LGE VPS and VTS demonstrated trend associations.

In the multivariable models (including age, sex, body mass index (BMI), LV ejection fraction (LVEF), LGE extent (%) using the FWHM method was an incremental outcome predictor (see Table 4). This also held true, when adding either the variable acute/subacute presentation to the multivariable model (LGE FWHM HR: 1.04, 95%CI: 1.00–1.08, *p* = 0.021) or the difference magnet strengths (1.5 and 3.0) (LGE FWHM HR 1.04, 95%CI 1.01–1.08, *p* = 0.016). Similarly, LGE extent (%) using the 2-SD method was independently associated with outcome (see Table 4 model 2), whereas 3-SD to 7-SD methods were not independent outcome predictors (all *p* = NS). Using visual scores, LGE-VPS was an independent predictor for outcome (see Table 4, model 5). This also held true when adding the variable acute/subacute presentation in the multivariable model to LGE-VPS (LGE-VPS HR: 1.06 (95%CI: 1.01–1.12, *p* = 0.015).

**Table 4:** Multivariabale Analysis of Association of LGE for MACE

Model 1			Model 2		
*Variables*	*HR (95% CI)*	*p-value*	*Variables*	*HR (95% CI)*	*p-value*
Age (years)	1.02 (1.01–1.04)	0.001	Age (years)	1.03 (1.01–1.04)	0.001
Sex	1.73 (1.15–2.60)	0.009	Sex	1.77 (1.17–2.65)	0.007
BMI	1.05 (1.02–1.08)	0.002	BMI	1.05 (1.02–1.08)	0.002
LVEF	0.96 (0.95–0.97)	< 0.001	LVEF	0.96 (0.95–0.97)	< 0.001
**LGE (%, FWHM)**	**1.04 (1.01–1.08)**	**0.009**	**LGE (%, 2-SD)**	1.02 (1.00–1.04)	**0.035**
Model 3			Model 4		
*Variables*	*HR (95% CI)*	*p-value*	*Variables*	*HR (95% CI)*	*p-value*
Age (years)	1.03 (1.01–1.04)	0.001	Age (years)	1.02 (1.00–1.04)	0.001
Sex	1.74 (1.16–2.62)	0.008	Sex	1.74 (1.12–2.61)	0.008
BMI	1.05 (1.02–1.08)	0.002	BMI	1.05 (1.02–1.08)	0.003
LVEF	0.96 (0.95–0.97)	< 0.001	LVEF	0.96 (0.95–0.97)	< 0.001
**LGE (%, 3-SD)**	**1.02 (0.99–1.05)**	**0.117**	**LGE (%, 4-SD)**	**1.02 (0.98–1.05)**	**0.309**
Model 5			Model 6		
*Variables*	*HR (95% CI)*	*p-value*	*Variables*	*HR (95% CI)*	*p-value*
Age (years)	1.02 (1.00–1.04)	0.001	Age (years)	1.02 (1.01–1.04)	0.002
Sex	1.74 (1.16–2.61)	0.008	Sex	1.70 (1.13–2.55)	0.010
BMI	1.05 (1.02–1.08)	0.002	BMI	1.05 (1.02–1.08)	0.002
LVEF	0.96 (0.95–0.97)	< 0.001	LVEF	0.96 (0.95–0.97)	< 0.001
**LGE (VPS)**	**1.07 (1.02–1.13)**	**0.005**	**LGE (VTS)**	1.01 (1.00–1.03)	**0.087**
Model 7					
*Variables*	*HR (95% CI)*	*p-value*			
Age (years)	1.02 (1.01–1.04)	0.005			
Sex	1.79 (1.19–2.70)	0.005			
BMI	1.05 (1.02–1.08)	0.001			
LVEF	0.96 (0.95–0.98)	< 0.001			
**LGE presence**	**1.82 (1.17–2.81)**	**0.008**			

*SD* Standard deviation, *LGE* late gadolinium enhancement, *LGE-VPS* LGE visual presence score, *LGE-VTS* LGE visual transmurality score

When adjusted to age, sex, BMI and LVEF, LGE-VPS maintained its strong association with MACE. For methods that quantified LGE size, LGE (%) by FWHM maintained robust adjusted association with MACE. In the multivariable model (including age, sex, BMI, LVEF; LGE extent and pericardial effusion), LGE extent (%, FWHM method) maintain significant adjusted association with MACE (HR 1.04. 95%CI: 1.01–1.08, *p* = 0.017), whereas pericardial effusion did not maintain its significance in the model (HR 1.5, 95%CI 0.96–2.3, *p* = 0.073). LGE image quality did not have an impact on the predictive value of LGE quantification methods.

**Fig. 4:**
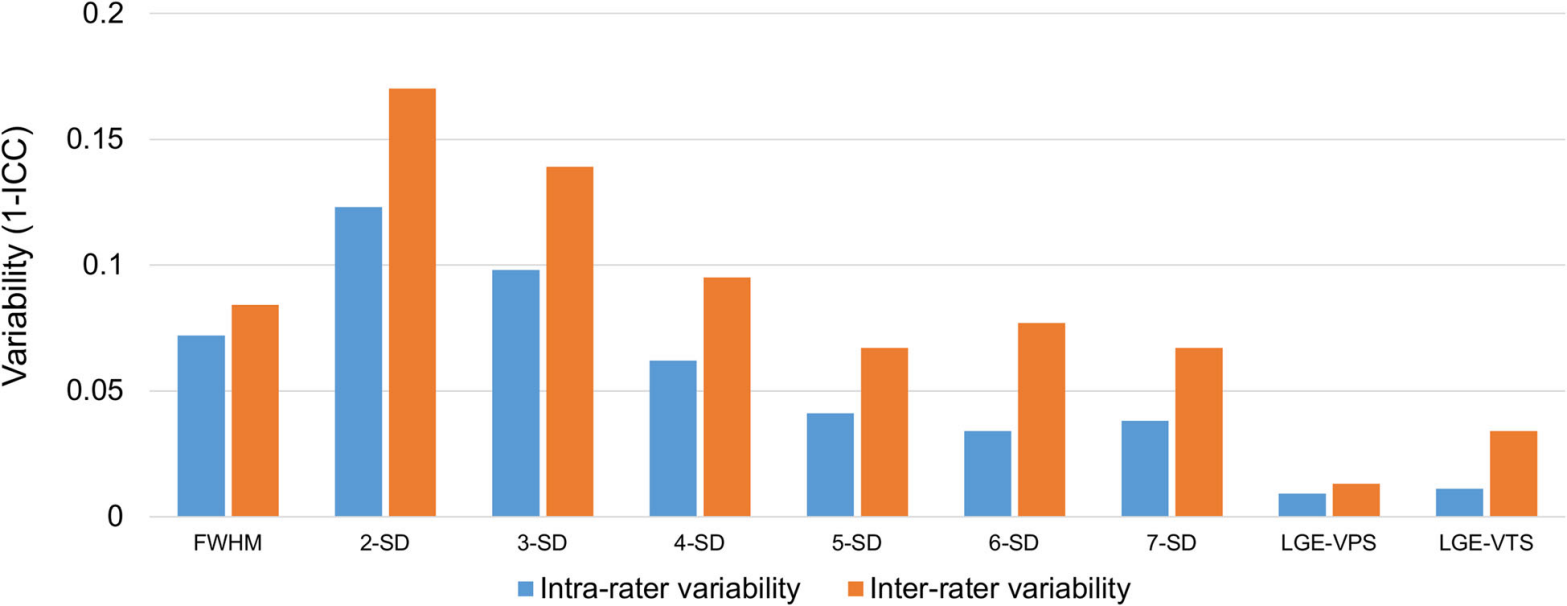
Reproducibility of different LGE quantification methods. Intra-rater and inter-rater variability for each LGE quantification method (calculated as 1 - intraclass correlation coefficient [ICC]) of the different methods is lowest in visual scores and FWHM. Intra-rater variability is less marked than inter-rater variability, as would be expected. Of the quantifications methods significantly associated with MACE, FWHM, LGE-VPS and LGE-VTS showed the best inter- and intra-rater variability. FWHM = Full width half maximum, SD = Standard deviation; LGE-VPS = visual LGE presence score; LGE-VTS = visual LGE transmurality score; MACE = Major adverse cardiovascular event

In the quantification methods significantly associated with outcome, excellent intra-rater and inter-rater reproducibility was achieved in visual scoring (LGE-VPS, LGE-VTS) and FHWM (*p* < 0.001) (see Fig. 4).

## Discussion

Our current study represents a systematic assessment of different semi-automated quantification methods of LGE extent in a large cohort of patients with suspected myocarditis. Our results based on adjusted Cox regression analyses and measurements of the degrees of intra-and inter-rater measurement reproducibility showed that FWHM demonstrated the highest technical consistency and the strongest prognostic association with MACE amongst all LGE quantification methods in patients with suspected myocarditis. On the other hand, visual qualitative assessment of LGE extent using the LGE-VPS score, represents a reliable alternative prognosticating method with an excellent intra-and inter-rater variability.

### LGE extent and outcome association

As consistent with other studies including myocardial infarction and hypertrophic cardiomyopathy patients, different LGE extent quantification methods result in a different extent of myocardial fibrosis, suggesting that the different methods are not interchangeable [13, 18]. This may have practical implications: thresholds that used lower SD cutoff may overestimate the extent of the LGE but possess higher sensitivities in detecting abnormal LGE. LGE-assessed myocardial fibrosis has been shown to be a predictor for outcome in patients with myocarditis [2, 8]. In fact, we could recently demonstrate that beside LVEF, LGE presence was associated with MACE [9], which is in line with a study including high risk myocarditis patients, where presence of LGE was an independent predictor of all-cause mortality [2]. To date, LGE extent proved to be an outcome predictor in patients with myocardial infarction, hypertrohpic cardiomyopathy and non-ischemic cardiomyopathy [14, 19–22]; however, no data based on larger studies were available on prognosis of different LGE extent quantification methods in suspected myocarditis patients.

Interestingly, in the multivariable model in our study only FWHM and 2-SD showed an independent association with outcome, but not using the 3 to 7-SD methods. Compared to myocardial infarction patients where higher SDs are used, lower SD might capture more substrate at risk (such as edema, fibrosis and diffuse scar) in myocarditis patients. Further, in the setting of myocarditis, the areas of LGE usually demonstrate lower signal intensity compared with LGE in patients with myocardial infarction. Therefore, based on the mean signal of the remote myocardium, and the difference between remote and pathological myocardium being smaller, higher SDs (especially 4 to 7-SD) might shift the threshold for LGE far from the remote myocardium and consequently underestimate the severity and extent of LGE. In studies including myocardial infarction patients, where LGE is commonly found bright, and apart from FWHM [13, 15], only thresholds higher than 2-SD (i.e. 3-SD to 6-SD) were proposed as accurate methods [23, 24]. However, in the setting of infarction, the different methods were not addressed with outcome association [25]. Interestingly our visual scoring showed strong association with outcome. Similar results were obtained from a small study only including 41 patients, where LGE extent (quantified by visual transmurality score, similar to our visual scores) remained an independent predictor of MACE with HR 1.42 [26]. Likewise, in another very small study from Barone-Rochette et al. including 28 patients with suspected myocarditis, a simplified quantitative score (SQS), similar to our LGE-VTS score, a trend towards worse outcome in those with higher initial SQS score (*p* = 0.08) could be shown [27]. It seems that visual assessment of LGE offers a rapid alternative for risk stratification in a diffuse or patchy disease such as myocarditis, where in contrast to myocardial infarction, more myocardium at risk might be involved than only territories subtended by a certain vessel.

### Inter-rater and intra-rater variability

Some authors used manual quantification as a reference standard to compare different quantification methods of LGE from myocardial infarction. In our opinion, manual contouring of LGE extent might be less consistent than semi-automatic quantitative methods in sizing LGE of patients with myocarditis. Compared to myocardial infarction, LGE from myocarditis tends to show more blurry contours and less intense enhancement. For example, in HCM patients presenting with rather patchy or diffuse LGE, manual delineation had poorer inter-and intra-rater variability compared to infarction patients [13], proving the manual technique not to be applicable in diseases with other LGE patterns than infarction. The heterogeneous nature of LGE presentations leads to a broad intra- and inter-rater variability and FWHM is more robust than the lower SDs in our study. In the quantification techniques associated with outcome, inter-rater and intra-rater variability showed highest reproducibility in the semi-automated FWHM technique, which was also shown in the mentioned study and by others likewise in different cardiac diseases [13, 18, 28, 29]. The FWHM method is less prone to over- or under-estimation in myocarditis patients, where LGE extent also might be represented by inflammation. This highlights the fact that different LGE quantification techniques are not necessarily equally applicable between different diseases since signal intensities are not comparable with those in infarction patients [13].

Another issue in myocarditis patients is that a high proportion present with subepicardial fibrosis [30] and in these cases epicardial contouring and delineation of epicardial LGE from epicardial fat, which also presents with a bright signal in LGE images, might pose issues for reproducibility. Therefore, even a small change in the epicardial contour might have a large impact on the intra- and inter-rater variability. Consequently, our visual scores – LGE-VPS and LGE-VTS (presence or absence of LGE or LGE transmurality) – may be less prone to variation and showed the highest inter-rater and intra-rater variability, which is consistent with prior literature [27].

### Limitations

There are several limitations for our study. This study is a retrospective observational design at a single center. Including patients evaluated on both 1.5 and 3.0 Tesla systems may potentially affect the LGE quantification; however, to the best of our knowledge, this effect is minimal on LGE sizing and in our multivariable model this had no significant impact on the results. There is no clinical reference standard for the assessment of LGE, so accuracy across the methods cannot be determined. In that regard, we chose to determine association with outcome as an alternative yet clinically relevant analysis. Our initial hypothesis was to evaluate which semi-quantitative method was best associated with the outcome. As an alternative method, we also evaluated the visual scoring. Our observations showed that both the visual LGE score and LGE quantitation using FWHM demonstrated robust prognostic values and measurement reproducibility, thus suggest that visual LGE score is a reliable clinical reporting parameter whereas FWHM be the signal intensity criteria of choice should LGE quantitation be needed. Our study was not designed to determine if image quality should be an additional factor in this decision in choosing these parameters, but our general impressions is that visual LGE scoring is more consistent. Similar to many clinical studies, details surrounding the immediate cause of death was not possible to obtain in a minority of patients, so we needed to rely on the use of all-cause mortality. Further, the use of immunosuppressant medication like steroids was not assessed, and therefore the influence of such medication on the outcome cannot be evaluated in this study. Finally, the natural course of acute myocarditis usually is followed by a decline in inflammation and LGE signal intensity changes during the stages of tissue healing. Although the inclusion of the variable acute/subacute presentation to the multivariable model did not change the results, future prospective studies will need to determine the need for optimization of the quantitative thresholds at different clinical settings (acute, subacute, delayed, chronic).

## Conclusions

LGE presence is a strong risk marker in patients with suspected myocarditis but quantitative methods of LGE sizing can offer a complementary and objective risk assessment. Amongst quantitative methods, LGE extent using FWHM criteria offers the highest prognostic value and high measurement reproducibility. Visual LGE scoring method is a reliable alternative.

## Abbreviations

BMI: Body mass index
bSSFP: Balanced steady state free precession
CAD: Coronary artery disease
CMR: Cardiovascular magnetic resonance imaging
CPK: Creatine phosphokinase
ECG: Electrocardiogram
eGFR: Estimated glomerular filtration rate
FWHM: Full width at half maximum
ICC: Intraclass-correlation coefficient
IQR: Interquartile range
LGE: Late gadolinium enhancement
LV: Left ventricle/left ventricular
MACE: Major adverse cardiovascular event
ROI: Region of interest
RV: Right ventricle/right ventricular
SD: Standard deviation
SQS: Simplified quantitative score
VPS: Visual (LGE) presence score
VTS: Visual (LGE) transmurality score
